# Candida albicans Adherence to Glass Ionomer Restorative Dental Material

**DOI:** 10.5681/joddd.2009.012

**Published:** 2009-06-05

**Authors:** Shirin Lawaf, Arash Azizi

**Affiliations:** ^1^Assistant Professor, Department of Prosthodontics, Faculty of Dentistry, Ahwaz Jundi Shapoor University of Medical science, Ahvaz, Iran; ^2^Associate professor, Department of oral Medicine, Faculty of Dentistry, Ahwaz Jundi Shapoor University of Medical sciences, Ahvaz, Iran

**Keywords:** Candida, whole saliva, glass ionomer, dental material

## Abstract

**Background and aims:**

It is believed that adherence of Candida albicans to oral surfaces is a critical event in the coloni-zation and development of oral diseases such as candida-associated denture stomatitis. Although there is considerable infor-mation about the adherence of Candida albicans to buccal epithelial cells and prosthetic materials, there is very little infor-mation available about the adherence of Candida albicans to glass ionomer materials. The purpose of this study was to investigate the degree of Candida albicans adherence to glass ionomer restorative material.

**Materials and methods:**

In this experimental study adherence of Candida albicans strains was studied with and without human whole saliva. First, glass ionomer fragments were prepared; then yeast cells were inoculated and incubated with differ-ent incubation times. After incubation, the fragments were removed from the wells and stained with 0.1% calcofluor white. Adhesion was quantified by counting the total number of cells at 40, 80 and 120 minutes. The analysis of variance and Stu-dent's test were used to assess the significance of differences between the means.

**Results:**

In the absence of saliva, the adherence of Candida albicans showed an increase, reaching a maximum at the end of the experiment (120 minutes). However, in the presence of saliva, the adherence of Candida albicans to glass ionomer significantly decreased.

**Conclusion:**

The presence of human whole saliva is an important factor in the adherence of Candida albicans to glass ion-omer restorative material.

## Introduction

Candidiasis is an opportunistic fungal infection most commonly caused by the Candida genus, Candida albicans.^1^ Approximately 60% of healthy adults and 45% to 65% of healthy children may harbor commensal candidal microorganisms without demonstrating any clinical signs or symptoms of mucosal disease.^2,3^ Under a variety of pathologic conditions, Candida species can proliferate in the mouth and produce oral lesions. Predisposing factors play a significant role in the development of oral candidiasis. Low salivary function, poor oral  hygiene,^4^ removal of intraoral prostheses,^5^ chronic antibiotic therapy,^6^ diabetes,^7^ systemic steroid therapy,^8^ and immunologic impairment (HIV infection)^9^ have been associated with increased susceptibility to oral candidiasis. Saliva plays a significant role in oral homeostasis. There is also some evidence that salivary IgA inhibits oral adhesion of Candida albicans.^10^

Medical implants such as catheters, prosthetic cardiac valves and dental prostheses have improved the health status of many patients. However, these devices can become colonized by different microorganisms to form a biofilm and establish a reservoir for chronic inoculation and dissemination of microbial cells. In the last few years, several studies have demonstrated the capacity of different microorganisms to adhere to tooth surfaces and dental prostheses. The surface hydrophobicity and electrostatic forces seem to play a key role in the adhesion process of Candida species to plastic materials.^11^ Maza showed that the presence of saliva is an important factor in the adherence of Candida albicans to resin-composite restorative materials.^12^Tronchin and Kennedy found that Candida albicans can adhere to plastic materials and predispose to Candida infections.^13,14^ Since adherence of oral microorganisms to restorative materials is a new area of research, there is not sufficient information regarding its role in the pathogenesis of oral diseases. However, it is expected that microorganisms adhering to restorative materials can colonize other oral surfaces, eventually causing oral infections in predisposed individuals. The purpose of this study was to investigate adherence of Candida albicans strains to glass ionomer restorative material because of its widespread clinical use. We have also studied the possible influence of human whole saliva on adherence, because we have recently demonstrated that saliva can play a role in the adherence of Candida albicans to polystyrene materials.^15^

## Materials and Methods

In this experimental study, a modified version of the method described by Tronchin et al^13^ was used. Unstimulated whole saliva samples were collected in the morning on the day the experiment was carried out from a healthy female donor to eliminate sample variations. The donor had not taken any medications during the 3-month period preceding the study and had no active periodontal disease or active caries or cigarette use.^13^ Saliva was kept at 4°C and used the same day. In order to prepare glass ionomer mixtures, equal ratios of powder and liquid of Fuji II LC glass ionomer (GC, Tokyo, Japan) were mixed according to manufacturer’s instructions.

Subsequently, the mixture was placed between 2 glass slides separated by spacers to leave a uniform distance of 1 mm between the glass slides. After photo-polymerization for 40 seconds from each side with a halogen light-curing unit (Coltolux 3, Coltene/Whaledent, Mahwah, NJ, USA), the plates were cut into fragments of 10×10 mm, polished with soft disks and washed with distilled water before being used in the adherence experiments. Yeast cells were inoculated into medium 199, pH 6.7 at a final concentration of 8 × 105 cells/mL and incubated for different times at 37°C in 24-well tissue culture polystyrene plates containing the glass ionomer pieces and 350 µL of the yeast cell suspension. After incubation, the fragments were removed from the wells, washed with saline solution, and stained with 0.1% calcofluor white in saline for 20 minutes at room temperature. Glass ionomer fragments were washed again, and the fluorescence was read under a microscope equipped to detect fluorescence. Adherence was quantified by counting the total number of cells at 40, 80, and 120 minutes, time intervals which present a wide range of adherence levels.^12^ For each glass ionomer square, 12 fields (0.64 mm^2^each) were counted by means of a graticule mounted in the focus of the ocular. Results were expressed as the number of cells per millimeter squared (mean values derived from 4 independent assays). All the values quoted represent mean figures derived from at least 4 independent assays. To determine the effect of saliva on adherence, the glass ionomer pieces were pre-incubated in 350 µL of whole saliva for 30 minutes at 37°C. T-test was used to compare the groups. Statistical significance was defined at P < 0.05.

## Results

**Figure 1:**
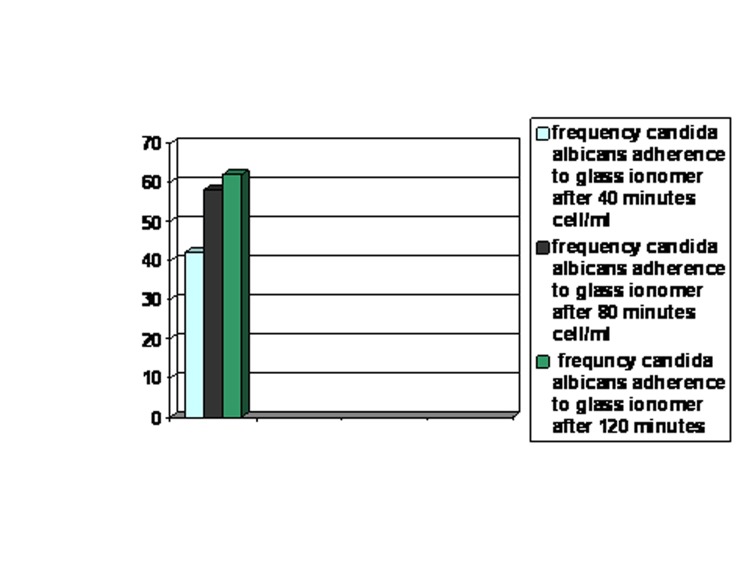
Adherence of Candida albicans to glass ionomer fragments in the absence of whole saliva.

**Figure 2:**
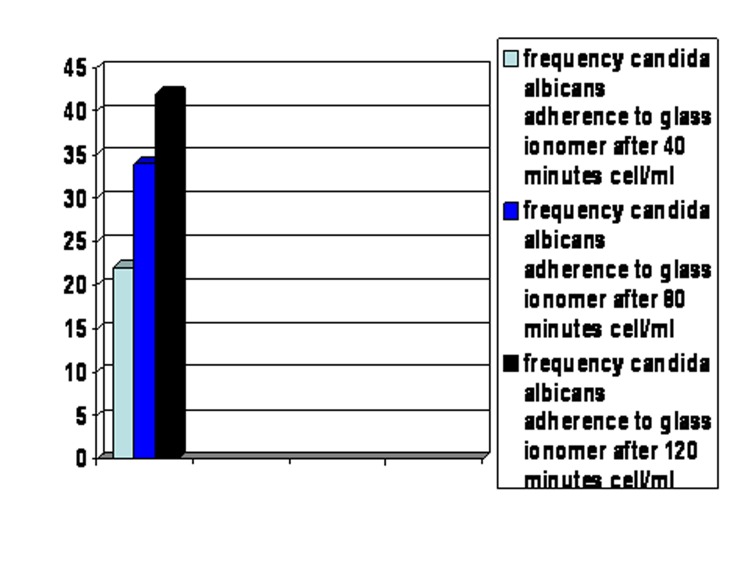
Adherence of Candida albicans to glass ionomer fragments in the presence of whole saliva.

In the absence of human saliva, the adherence of Candida albicans to glass ionomer increased with time, reaching a maximum at 120 minutes (Figure 1). In the presence of saliva, the adherence of Candida albicans to glass ionomer also increased with time reaching a maximum at 120 minutes (Figure 2). Saliva significantly reduced the adherence of Candida albicans to glass ionomer fragments when compared with the adherence in the absence of saliva (P < 0.01).

The means of yeast cell adherence to glass ionomer fragments in the presence and absence of saliva were 22, 34, and 42 cells/mL at 40, 80, and 120 minutes, and 42, 58, and 62 cells/mL at 40, 80, and 120 minutes, respectively.

## Discussion

Infections caused by Candida species are increasing as the number of immunocompromised patients in the community increases. Thus, oral candidiasis is the most common oral opportunistic infection seen in such patients. Other risk factors for oral candidiasis include aging, pregnancy, denture wearing, poor oral hygiene, diabetes, antibiotic therapy and immunologic impairment. Although other Candida species may be involved, Candida albicans is the major etiologic agent in oral and systemic candidiasis.^16^ In addition, adherence of Candida albicans to artificial materials such as those of dental prostheses, catheters and other medical devices seems to be a critical event in the initiation of colonization and infection. This process may be specially important in denture stomatitis, where Candida albicans can adhere to the acrylic resin to form a reservoir for chronic dissemination of fungal cells.^17^ Despite the information about the adherence of oral microorganisms to these materials, the published information is mainly restricted to oral bacteria.^18^ Our group is interested in the adherence of Candida species to glass ionomer. Some researchers have previously developed a model to study the adherence of Candida species to glass ionomer fragments.^19^ The results of the present study showed that Candida albicans can directly adhere to glass ionomer restorative material, which is consistent with previous results obtained using plastic materials.^13,14,20^ Studies have found that Candida albicans can adhere to plastic and composite materials and initiate colonization of candidal infections.^12 -14 ,20^

Since the adherence of Candida albicans to oral surfaces has been purported to be modulated by different salivary components, we have also included saliva in this study.

The effect of human whole saliva on the adherence of Candida albicans to glass ionomer fragments was evaluated. Under the conditions evaluated, whole saliva caused a statistically significant decrease in adherence. Our results are consistent with those of Samaranayake and MacFarlane^21^ and McCourtie et al,^22^ who have demonstrated that pre-incubation of acrylic strips with whole saliva decreases the adherence of Candida albicans to denture acrylic resin in vitro.

The salivary components responsible for the decrease in adherence of Candida albicans to glass ionomer fragments are presently unknown, but saliva contains different components including high molecular weight mucin, amylase, and secretory IgA, which have been shown to decrease adherence of Candida albicans to denture acrylic resin in vitro and to epithelial tumor cells.^21,23^

## Conclusion

The results of the present study demonstrated that the presence of human whole saliva is an important factor in the adherence of Candida albicans to the glass ionomer restorative material.

## References

[1] Crockett DN, O'grady JF, Reade PC (1992). Candida species and candida albicans morphotypes in erythematous candidiasis. Oral Surg Oral Med Oral Pathol.

[2] Fotos PG, Vincent SD, Hellstein JW (1992). Oral candidosis. clinical, historical, and therapeutic features of 100 cases. Oral Surg Oral Med Oral Pathol.

[3] Berdicevsky I, Ben-aryeh H, Szargel R, Gutman D (1984). Oral candida in children. Oral Surg Oral Med Oral Pathol.

[4] Wahlin YB (1991). Salivary secretion rate, yeast cells, and oral candidiasis in patients with acute leukemia. Oral Surg Oral Med Oral Pathol.

[5] Barbeau J, Séguin J, Goulet JP, De KONINCK L, Avon SL, Lalonde B, Et AL (2003). Reassessing the presence of candida albicans in denture-related stomatitis. Oral Surg Oral Med Oral Pathol Oral Radiol Endod.

[6] Beighton D, Hellyer PH, Lynch EJ, Heath MR (1991). Salivary levels of mutans streptococci, lactobacilli, yeasts, and root caries prevalence in non-institutionalized elderly dental patients. Community Dent Oral Epidemiol.

[7] Bánóczy J, Albrecht M, Rigó O, Ember G, Ritlop B (1987). Salivary secretion rate, ph, lactobacilli and yeast counts in diabetic women. Acta Diabetol Lat.

[8] Folb PI, Trounce JR (1970). Immunological aspects of candida infection complicating steroid and immunosuppressive drug therapy. Lancet.

[9] Tylenda CA, Larsen J, Yeh CK, Lane HC, Fox PC (1989). High levels of oral yeasts in early hiv-1 infection. J Oral Pathol Med.

[10] Challacombe S (1990). Immunology of oral candidiasis. In: Samaranayake LP, Macfarlane TW, eds. Oral Candidiasis.

[11] Klotz SA, Drutz DJ, Zajic JE (1985). Factors governing adherence of candida species to plastic surfaces. Infect Immun.

[12] Maza JL, Elguezabal N, Prado C, Ellacuría J, Soler I, Pontón J (2002). Candida albicans adherence to resin-composite restorative dental material: influence of whole human saliva. Oral Surg Oral Med Oral Pathol Oral Radiol Endod.

[13] Tronchin G, Bouchara JP, Robert R, Senet JM (1988). Adherence of candida albicans germ tubes to plastic: ultrastructural and molecular studies of fibrillar adhesins. Infect Immun.

[14] Kennedy MJ, Rogers AL, Yancey RJ JR (1989). Environmental alteration and phenotypic regulation of candida albicans adhesion to plastic. Infect Immun.

[15] San MILLáN R, Elguezabal N, Regúlez P, Moragues MD, Quindós G, Pontón J (2000). Effect of salivary secretory iga on the adhesion of candida albicans to polystyrene. Microbiology.

[16] Garber GE (1994). Treatment of oral candida mucositis infections. Drugs.

[17] Shahal Y, Steinberg D, Hirschfeld Z, Bronshteyn M, Kopolovic K (1998). In vitro bacterial adherence onto pellicle-coated aesthetic restorative materials. J Oral Rehabil.

[18] Suljak JP, Reid G, Wood SM, Mcconnell RJ, Van DER MEI, Busscher HJ (1995). Bacterial adhesion to dental amalgam and three resin composites. J Dent.

[19] Maza JL, Prado C, Vidotto V, Elguezábal N, Pontón J (2001). Adherencia de diversas especies de candida a composites híbridos. Rev Eur Odontoestomatol.

[20] Rotrosen D, Edwards JE JR, Gibson TR, Moore JC, Cohen AH, Green I (1985). Adherence of candida to cultured vascular endothelial cells: mechanisms of attachment and endothelial cell penetration. J Infect Dis.

[21] Samaranayake LP, Macfarlane TW (1980). An in-vitro study of the adherence of candida albicans to acrylic surfaces. Arch Oral Biol.

[22] Mccourtie J, Macfarlane TW, Samaranayake LP (1986). A comparison of the effects of chlorhexidine gluconate, amphotericin b and nystatin on the adherence of candida species to denture acrylic. J Antimicrob Chemother.

[23] Umazume M, Ueta E, Osaki T (1995). Reduced inhibition of candida albicans adhesion by saliva from patients receiving oral cancer therapy. J Clin Microbiol.

